# Controlling the magnetic structure in W-type hexaferrites

**DOI:** 10.1107/S1600576723002133

**Published:** 2023-04-13

**Authors:** Mathias I. Mørch, Mogens Christensen

**Affiliations:** a Center for Materials Crystallography, Department of Chemistry and Interdisciplinary Nanoscience Center (iNANO), Aarhus Universitet, Langelandsgade 140, Aarhus C, 8000, Denmark; Australian Nuclear Science and Technology Organisation, Lucas Heights, Australia

**Keywords:** hexaferrites, magnetic ordering, neutron diffraction, multiferroics

## Abstract

W-type hexaferrites with varied Co/Zn ratios were synthesized and the magnetic order was investigated using neutron powder diffraction. In SrCo_2_Fe_16_O_27_ and SrCoZnFe_16_O_27_ a planar (*Cm*′*cm*′) magnetic ordering was found, rather than the uniaxial ordering (*P*6_3_/*mm*′*c*′) found in SrZn_2_Fe_16_O_27_ which is common in most W-type hexaferrites. In all three studied samples, non-collinear terms were present in the magnetic ordering. One of the non-collinear terms is common to the planar ordering in SrCoZnFe_16_O_27_ and uniaxial ordering in SrZn_2_Fe_16_O_27_, which could be a sign of an imminent transition in the magnetic structure. The thermomagnetic measurements revealed magnetic transitions at 520 and 360 K for SrCo_2_Fe_16_O_27_ and SrCoZnFe_16_O_27_, and Curie temperatures of 780 and 680 K, respectively, while SrZn_2_Fe_16_O_27_ showed no transition but a Curie temperature at 590 K. This leads to the conclusion that the magnetic transition can be adjusted by fine-tuning the Co/Zn stoichiometry in the sample.

## Abstract 2

Using neutron powder diffraction on W-type hexaferrites, planar magnetic ordering described in *Cm*′*cm*′ was found for SrCo_2_Fe_16_O_27_ and SrCoZnFe_16_O_27_, while SrZn_2_Fe_16_O_27_ had uniaxial ordering described in *P*6_3_/*mm*′*c*′. Thermomagnetic measurements above room temperature also indicated magnetic transitions for SrCo_2_Fe_16_O_27_ and SrCoZnFe_16_O_27_.

## 1. Introduction

Magnetoelectric materials are theorized to enable a plethora of new electronics. One of these is new energy-efficient electronics for data storage, which is important to combat the increasing energy demand from information and communications technologies (Andrae & Edler, 2015 ▸). Magnetoelectric materials employed in logic devices that employ spin–orbit logic have shown potential beyond complementary metal–oxide–semiconductor transistors in terms of switching energy, switching voltage and logic density (Manipatruni *et al.*, 2019 ▸). The magnetoelectric memory is also non-volatile, *i.e.* it does not need constant power to store the information.

Despite the promising applications of magnetoelectric materials, their property of poor magnetoelectric coefficients, or none at all, at room temperature is still a significant challenge. Magnetoelectric hexaferrites are a promising class of multiferroic materials that are attracting increasing interest (Kimura, 2012 ▸) because they hold promise for overcoming some of these challenges. One type of hexaferrite, the Z-type, has recently been demonstrated as a single-phase room-temperature non-volatile memory (Zhai *et al.*, 2018 ▸). Magneto­electric effects have been discovered across the hexaferrite family, where the Y-type hexaferrite Ba_2−*x*_Sr*_x_* Mg_2_Fe_12_O_22_ (Zhai *et al.*, 2017 ▸), the Z-type hexaferrite Sr_3_Co_3_Fe_24_O_41_ (Kitagawa *et al.*, 2010 ▸) and W-type hexaferrites (Song *et al.*, 2014 ▸) are worth noting. Common to all is the potential for controlling the magnetoelectric effect through structural and substitutional modifications.

It is widely accepted that the feature responsible for magnetoelectric coupling in hexaferrites is the presence of conical spin order (Nakajima *et al.*, 2016 ▸; Kocsis *et al.*, 2019 ▸; Zhai *et al.*, 2017 ▸; Shirokov *et al.*, 2021 ▸). These conical orderings give rise to magnetoelectric coupling resulting from anti-symmetric exchange: the inverse Dzylaoshinkii–Moriya interaction (Tokura *et al.*, 2014 ▸; Fabrykiewicz *et al.*, 2021 ▸).

A system with potential conical spin ordering is the family of W-type hexaferrites, with Co^2+^ substituting Fe^2+^ as the transition metal. It is known that Co^2+^ changes the magnetocrystalline anisotropy axis from uniaxial to planar at room temperature for SrCo_2−*x*_Zn*_x_* Fe_16_O_27_ when *x* goes from 2 to 0 (Mørch *et al.*, 2019 ▸). Here, three different magnetic structures of W-type hexaferrites are determined by neutron powder diffraction methods at room temperature. The compounds investigated are W-type hexaferrites, SrCo_2−*x*_Zn*_x_* Fe_16_O_27_ (*x* = 0, 1 and 2), and the samples are investigated by combined Rietveld refinement of a model to high-resolution neutron and X-ray powder diffraction data.

It is highly relevant to determine the magnetic structures of SrCo_2−*x*_Zn*_x_* Fe_16_O_27_ because each compound is a possible magnetoelectric material. Previous attempts to determine the magnetic structure have been limited to assigning the ferrimagnetic ordering of the seven sublattices and assessing whether the general ordering is planar, canted or uniaxial. These descriptions do not accurately represent the inherent symmetry of the material or lead to an understanding of the coupling between ferroelectricity and ferromagnetism. Magnetoelectric coupling mechanisms, such as the inverse Dzyaloshinskii–Moriya effect, necessitate the breaking of inversion symmetry. An accurate description of the structure, both magnetic and nuclear, can lead to an understanding that further improves current and new magnetoelectric materials.

To our knowledge, conical spin order in SrCo_2−*x*_Zn*_x_* Fe_16_O_27_ has been assumed but no investigation conducted. While BaCo_2−*x*_Zn*_x_* Fe_16_O_27_ have been investigated (Paoluzi *et al.*, 1988 ▸) and a conical order assumed, only one end member, BaCo_2_Fe_16_O_27_, was investigated using neutron diffraction up to a *Q*_max_ of 3.2 Å^−1^ [*Q* = (4π/λ)sin(θ/2), where θ is the scattering angle and λ is the wavelength of the incident radiation] with 11 measured reflections (100), (200) and [(002):(0018)]. The structure was reported to have an easy-plane total spin alignment, but no further discussion of the symmetry was given. This paper reports the different magnetic symmetries present in the W-type hexaferrites (WHFs) SrCo_2−*x*_Zn*_x_* Fe_16_O_27_ investigated by powder diffraction at room temperature. The ambient diffraction data are supported by isofield thermomagnetic measurements of samples in the range *x* = 0 to 2 in steps of 0.5, to support the understanding of a composition-dependent controllable magnetic transition temperature.

## 2. Experimental

### 2.1. Synthesis

SrCo_2−*x*_ZnFe_16_O_27_ was synthesized by a sol–gel autocombustion step. First, the salts Sr(NO_3_)_2_, Fe(NO_3_)_3_·9H_2_O and/or Co(NO_3_)_2_·6H_2_O and/or Zn(NO_3_)_2_·6H_2_O (all Sigma–Aldrich ACS reagent grade, ≥98.0% purity) were weighed in stoichiometric molar ratios and dissolved in 125 ml of de­mineralized water in a 2000 ml crystallizing dish. Citric acid was also dissolved in 125 ml of demineralized water and added in an equal ratio to the nitrates under constant stirring. The solution was neutralized with approximately 75 ml of NH_4_OH (≥25% NH_3_ alkaline solution) and dried overnight in a convection oven at 373 K until a gel was formed. Subsequently, the gel was fired in a preheated furnace at 623 K for 30 min until the autocombustion had finished and then cooled to room temperature in air. The dried and crushed powders were calcined in a furnace as indicated in Table 1 ▸. The optimal sintering temperature was found through multiple synthesis attempts at different temperatures. The chosen temperatures produced the most phase pure samples.

### 2.2. Powder diffraction

Both neutron powder diffraction data and synchrotron X-ray powder diffraction data were measured for the prepared samples. The high-quality high-resolution data were measured using the mail-in service on the following instruments.

Neutron powder diffraction data were measured for the three samples on the SuperHRPD beamline at J-PARC, Tokai-Mura, Ibaraka, Japan (Torii *et al.*, 2011 ▸, 2014 ▸). Samples were packed in vanadium cans and data were collected at room temperature using the time-of-flight (TOF) method on three detector banks. For the high-angle bank *Q* = 1.567–24.89 Å^−1^, for the 90° bank *Q* = 1.176–18.01 Å^−1^ and for the low-angle bank *Q* = 0.397–9.057 Å^−1^. Data were collected for 8 h from each sample.

Synchrotron X-ray powder diffraction data were measured on beamline I11 at Diamond Light Source, Didcot, Oxfordshire, UK (Thompson *et al.*, 2009 ▸). The wavelength was 0.826555 (10) Å and samples were packed in 0.3 mm glass capillaries. The data were collected at room temperature using Debye–Scherrer geometry employing multi-analysing crystal devices in a constant-velocity scan covering the angular range 2θ = 0–150° (data below 2° were blocked by the beam stop), *Q* = 0.53–14.7 Å^−1^. The total collection time was 1 h per sample.

Figs. S1 and S2 in the supporting information show the four collected diffraction patterns and the refined model for SrCo_2_Fe_16_O_27_. In addition to this, CIFs containing all refined patterns were generated using *TOPAS* macros as described in *pdCIFplotter* (Rowles, 2022 ▸), which also describes software to visualize the patterns; these files are available as a ZIP archive in the supporting information.

### 2.3. Magnetic measurements

The magnetic measurements were carried out with a Physical Properties Measuring System (PPMS) from Quantum Design equipped with a vibrating sample magnetometer with an oven attachment. Powder samples were dispersed in high-temperature zircar cement (product number QDS-4097-030), and a small nugget was dried on top of the resistive platinum heater. A copper foil was wrapped around the sample and heater to minimize radiative heat loss. Isofield thermomagnetic measurements were carried out for all three samples, where magnetization versus temperature was measured from 300 to 900 K. For all measurements the heating and cooling rate was 10 K min^−1^. Before each initial heat ramp, samples were magnetized at 10 000 Oe (797.70 kA m^−1^). The five samples SrCo_2−*x*_Zn*_x_* Fe_16_O_27_ (*x* = 0, 0.5, 1, 1.5 and 2) were measured upon heating, with an applied field of 100 Oe (7.98 kA m^−1^) for SrCo_2_Fe_16_O_27_ and 50 Oe (3.99 kA m^−1^) for the remainder.

## 3. Results and discussion

The powder diffraction experiments carried out at room temperature show the two different magnetic orderings at play, while the isofield thermomagnetometry experiments indicate when, where and how this ordering might change. For an accurate description of the structural and magnetic differences it is also highly important to characterize correctly any impurities present.

### 3.1. Refinement details

Rietveld refinements of the crystal and magnetic structures were carried out using *TOPAS* (Version 6; Coelho, 2018 ▸) where the four patterns (one X-ray and three neutron TOF, one per bank) for a given composition were refined in a constrained refinement. Symmetry mode analysis was carried out using *ISODISTORT* (part of the *ISOTROPY Software Suite*, https://iso.byu.edu/iso/isotropy.php) to generate the magnetic structures for the three samples. The number of independent observations was calculated for each as described by David (1999 ▸), giving ratios of independent observations to parameters affecting peak intensity of the crystal and magnetic structures of 28:1 and 11:1, respectively. An overview of agreement factors is given in Table 2 ▸. For further details, see Section S1.2. All full diffraction patterns are also available in the supporting information.

### 3.2. Magnetic structure

**Figure 1:**
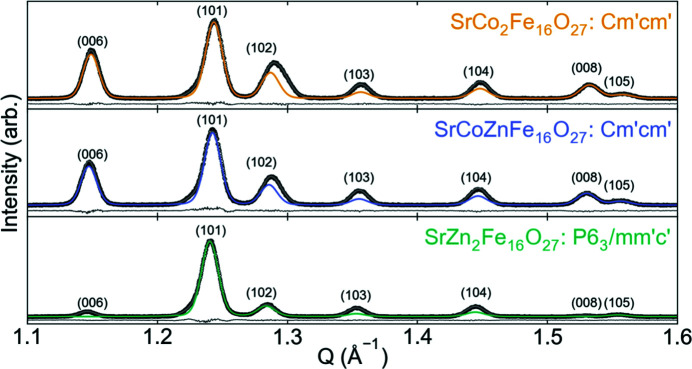
Powder diffraction patterns from the SHRPD forward scatter bank for the three samples. *I*_obs_ are shown as black circles, *I*_calc_ as grey lines and *I*_obs−calc_ as the black lines offset under each plot. The magnetic scattering contribution is highlighted with the respective colour for each sample. (*hkl*) indices are shown above the peaks, and indices for SrCo_2_Fe_16_O_27_ and SrCoZnFe_16_O_27_ are also given in *P*6_3_/*mmc* for simplicity. Near the (102) peak is the (101) spinel impurity peak. The main contribution to this peak is also magnetic, explaining why it is not observed in SrZn_2_Fe_16_O_27_ as the spinel impurity ZnFe_2_O_4_ has no long-range magnetic order.

In our previous study we described the uniaxial magnetic ordering in SrZn_2_Fe_16_O_27_ with the magnetic space group (MSG) *P*6_3_/*mm*′*c*′ (further details given in Section S2.1), but did not determine the MSG of the planar ordering in SrCo_2_Fe_16_O_27_ (Mørch *et al.*, 2019 ▸). Fig. 1 ▸ shows highlights of the refined magnetic contribution to the powder diffractograms for the three samples. The presence of the stronger (006) peak at 1.15 Å^−1^ in SrCo_2_Fe_16_O_27_ and SrCoZnFe_16_O_27_ indicates magnetic ordering perpendicular to the *c* axis, as the magnetic scattering is given by 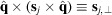 (Lefmann, 2017 ▸; Marshall & Lovesey, 1971 ▸). Here, 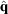 is the scattering vector, while, **s**_*j*_ is the spin on site *j* and 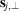 is the spin on site *j* perpendicular to the scattering vector. As the materials are ferrimagnetic, we further limit the search to MSGs which have ferromagnetic ordering on all Wyckoff sites occupied by Fe. *Cm*′*cm*′ and *Cmc*′*m*′ are both maximal subgroups of *P*6_3_/*mmc*1′ that fulfil this criterion of magnetic ordering in the *ab* plane giving rise to ferromagnetism. Fig. S3 shows the relation between *P*6_3_/*mmc* and *Cmcm*. We chose *Cm*′*cm*′ to describe the data as the concurrently active mGM4+ mode describes a significant canting towards the *c* axis (further described in Section 3.4).

**Figure 2:**
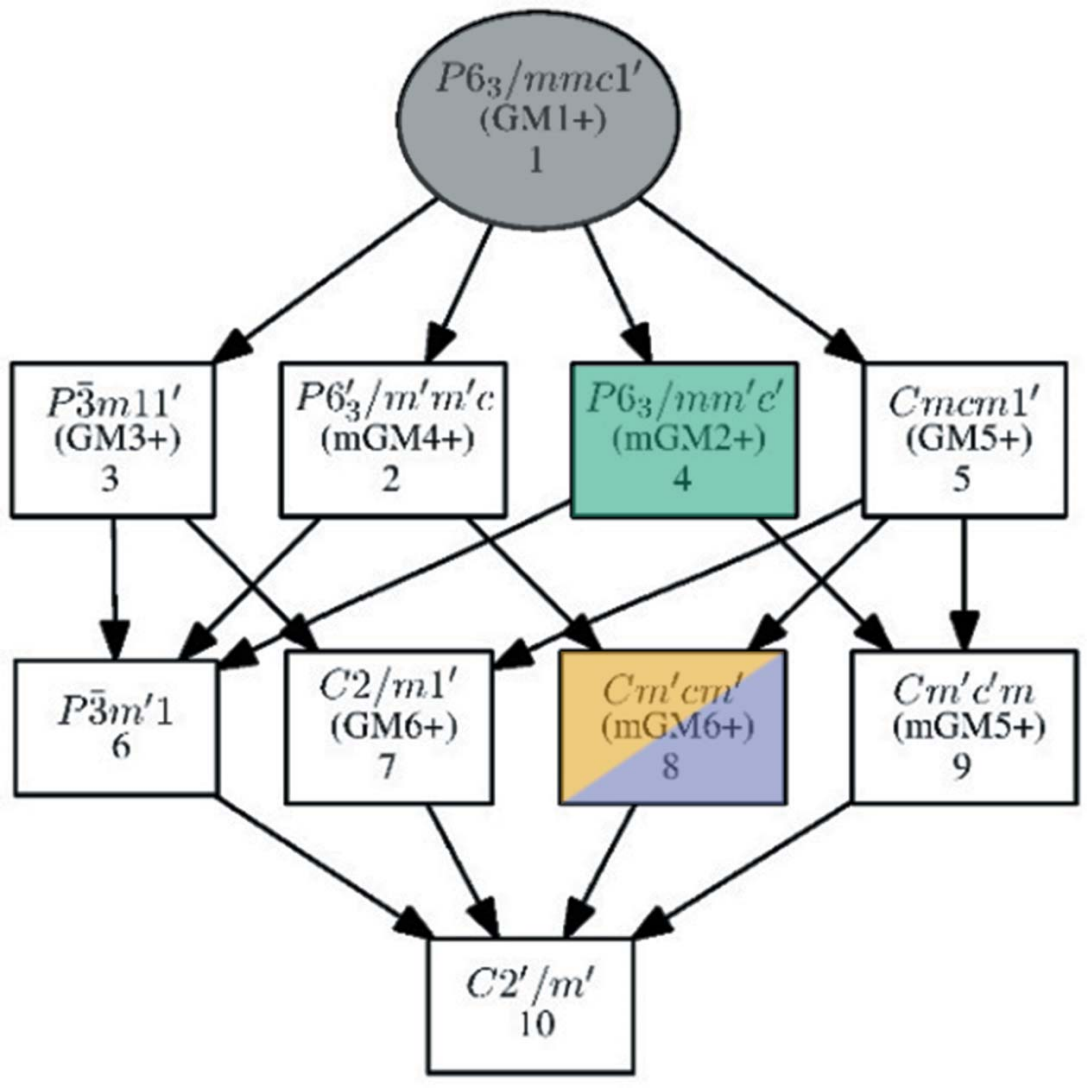
Group–subgroup relations of the paramagnetic parent *P*6_3_/*mmc*1′, the uniaxial ferrimagnet *P*6_3_/*mm*′*c*′, the planar ferrimagnet *Cm*′*cm*′ and the hypothetical angled collinear ferrimagnet *C*2′/*m*′. Note that no direct group–subgroup relation relates to the uniaxial and planar orderings. Graph created using the tool *Get_mirreps* (Perez-Mato *et al.*, 2015 ▸) on the Bilbao Crystallographic Server (Aroyo, Kirov *et al.*, 2006 ▸; Aroyo, Perez-Mato *et al.*, 2006 ▸; Aroyo *et al.*, 2011 ▸).

Fig. 2 ▸ shows the relation between the paramagnetic parent *P*6_3_/*mmc*1′ and the two refined models, *P*6_3_/*mm*′*c*′ and *Cm*′*cm*′. A possible common subgroup, *C*2′/*m*′, for these is also shown. Fig. S5 shows the relation between the paramagnetic parent *P*6_3_/*mmc*1′ and the alternative *Cmc*′*m*′ and *P*6_3_/*mm*′*c*′. The MSG *P*6_3_/*mm*′*c*′ only allows for ferromagnetism along the *c* axis, while in the *Cm*′*cm*′ MSG ferromagnetism in the *ab* plane is allowed. The MSG *C*2′/*m*′ is of low enough symmetry to allow for ferromagnetic order at any angle to the *c* axis.

### 3.3. Occupancy and purity

**Figure 3:**
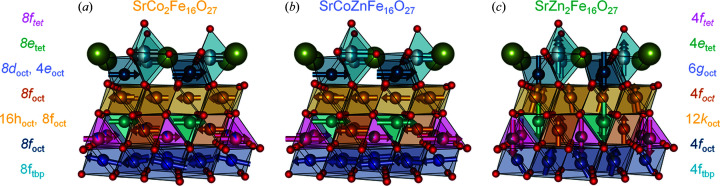
Illustrations of the magnetic ordering in (*a*) SrCo_2_Fe_16_O_27_, (*b*) SrCoZnFe_16_O_27_ and (*c*) SrZn_2_Fe_16_O_27_. Shown from *c* = 0:0.25, and SrZn_2_Fe_16_O_27_ extended to the orthorhombic basis used for SrCo_2_Fe_16_O_27_ and SrCoZnFe_16_O_27_.

Good contrast between Co, Fe and Zn in the combined refinement allowed for a robust refinement of the respective occupancies. All sites were assumed to be fully occupied, while Co and Zn occupancies were refined on all sites. For SrCoZnFe_16_O_27_ where both Co and Zn could substitute for Fe, Fe_Occ_ and Zn_Occ_ were refined, with Co given as Co_Occ_ = 1 − Fe_occ_ − Zn_Occ_. As a consequence of reducing the symmetry from *P*6_3_/*mm*′*c*′ to *Cm*′*cm*′, some Wyckoff sites were split. Co primarily occupied the split 8*d*_oct_ and 4*e*_oct_ sites, with a preference for 8*d*_oct_ in SrCo_2_Fe_16_O_27_ and for 4*e*_oct_ in SrCoZnFe_16_O_27_ (Fig. 3 ▸). This shows that assuming equal site occupancy behaviour between compositions is not necessarily correct, which could be revealed here due to the good Fe/Co contrast available. Zn primarily occupied the 8*f*_tet_ (4*f*_tet_) site and then the 8*e*_tet_ (4*e*_tet_) site. The primary occupancy sites of Co on octahedral sites and Zn on tetrahedral sites follow the expected inversion behaviour from spinels, as previously described by Mørch *et al.* (2019 ▸). The resulting compositions from the refinements are SrCo_1.71 (3)_Fe_16.29 (3)_O_27_, SrCo_0.84 (9)_Zn_0.81 (13)_Fe_16.35 (9)_O_27_ and SrZn_1.799 (11)_Fe_16.201 (11)_O_27_.

The high-quality data also made it possible to account for impurities, for which more information is given in Table 2 ▸. A full overview of the refined occupancies, atomic positions and atomic displacement parameters is given in the supporting information in Tables S1–S3.

### 3.4. Magnetic ordering

The magnetic refinement was set up as a distortion-mode refinement in *TOPAS* by exporting the TOPAS.str file from *ISODISTORT*. The symmetry-mode refinement makes it possible to turn on/off non-collinear components easily to investigate their significance. The magnitude of the distortion was compared with the uncertainty, and it was found that all three samples had non-collinear magnetic symmetry modes active. Sites with partial Fe/Co occupancy had the relative magnitude of the magnetic moment fixed to the number of unpaired electrons (5:3). The planar magnetic ordering of SrCo_2_Fe_16_O_27_ and SrCoZnFe_16_O_27_ is well described in the magnetic space group *Cm*′*cm*′ using the irreducible representations mGM6+ and mGM4+. Canting towards the *c* axis with antiferromagnetic ordering is observed predominantly on the 8*f*_tet_, 8*f*_oct_ and 8*f*_tbp_ sites. In SrCoZnFe_16_O_27_ an additional small antiferromagnetic ordering in the *ab* plane is observed for the 8*d*_oct_ site.

The axial ordering of SrZn_2_Fe_16_O_27_ is well described using mGm2+ in the MSG *P*6_3_/*mm*′*c*′. A small canting towards the *ab* plane, with antiferromagnetic ordering, is observed for the 6*g*_oct_ and 12*k*_oct_ sites. In Fig. 3 ▸ models of the three magnetic structures are shown. Additional details of the magnetic structures are given in Tables S1–S3.

As noted by Shirokov *et al.* (2021 ▸), a ‘truly’ collinear magnetic ordering is not symmetry protected in either *P*6_3_/*mm*′*c*′ or *Cm*′*cm*′, and the model best describing our data supports the inclusion of these non-collinear terms. The observation of similar canting on the 8*d*_oct_ site in SrCoZnFe_16_O_27_ and the 6*g*_oct_ site in SrZn_2_Fe_16_O_27_ (highlighted in Fig. S5), while absent in SrCo_2_Fe_16_O_27_, could indicate the preliminary stages of a transition in the magnetic structure.

### 3.5. Magnetic transition

**Figure 4:**
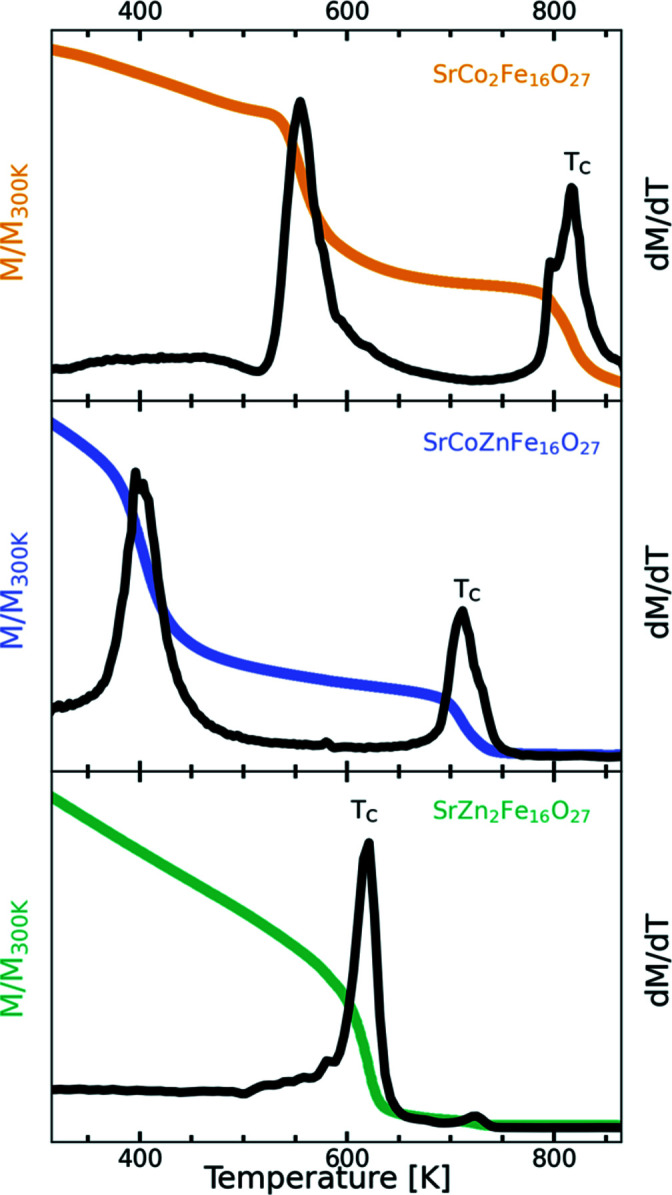
Thermomagnetic measurements of SrCo_2_Fe_16_O_27_, SrCoZnFe_16_O_27_ and SrZn_2_Fe_16_O_27_. The applied fields were, respectively, 100, 50 and 50 Oe. The temperature range covered was 300–900 K, while the shown range is limited to 325–875 K. The magnetization (in colour) and a derivative thereof (in black) are normalized versus the magnetization at 300 K as *M*/*M*_300 K_ for easy comparison. Indications of the onset of a magnetic transition (a peak in the derivative lower than the Curie temperature) are seen for SrCo_2_Fe_16_O_27_ at 520 K and SrCoZnFe_16_O_27_ at 360 K.

Isofield thermal magnetometry has previously been used to show the transition between uniaxial, planar and conical magnetic orderings in hexaferrites (Takada *et al.*, 2005 ▸; Tachibana *et al.*, 2003 ▸). Here, the thermomagnetic data shown in Fig. 4 ▸ are for *x* = 0, 1 and 2 and reveal magnetic transitions well below the Curie temperature for both SrCo_2_Fe_16_O_27_ and SrCoZnFe_16_O_27_, seen as a steep decrease in magnetization with increasing temperature. This feature is observed around 520 K for SrCo_2_Fe_16_O_27_ and 360 K for SrCoZnFe_16_O_27_ but is not present in SrZn_2_Fe_16_O_27_. The weak transition visible around ∼725 K in SrZn_2_Fe_16_O_27_ is near the Curie temperature of the SrFe_12_O_19_ impurity at ∼750 K (Shirk & Buessem, 1969 ▸). The changes in transition temperature between the samples indicate that the transition can be tuned by adjusting the Co/Zn ratio.

In addition to the three samples investigated using neutron diffraction, thermomagnetic measurements of SrCo_1.5_Zn_0.5_Fe_16_O_27_ and SrCo_0.5_Zn_1.5_Fe_16_O_27_ were also conducted. The transition temperatures indicated by the maximum of the derivative along with the Curie temperatures were extracted and are given in Table 3 ▸. From the data, it is also seen that the Curie temperature decreases with increasing Zn content.

**Figure 5:**
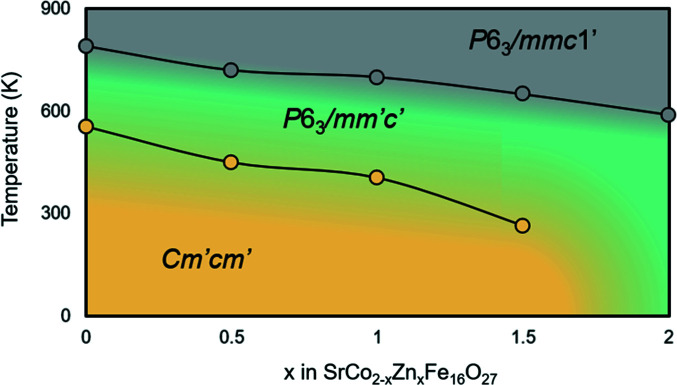
A tentative magnetic phase diagram for SrCo_2−*x*_Zn*_x_* Fe_16_O_27_

By combining the results from the refinement of the magnetic structures with the thermomagnetic measurements, a tentative magnetic phase diagram can be constructed (Fig. 5 ▸). A previous study by Graetsch *et al.* (1984 ▸) also indicated a change in the magnetocrystalline anisotropy for composition and temperature. A sample with a composition around SrCo_0.65_Zn_1.35_Fe_16_O_27_ would have a magnetic phase transition between *Cm*′*cm*′ and *P*6_3_/*mm*′*c*′ at room temperature, possibly giving rise to interesting magnetic properties including magnetoelectrics at room temperature.

To clarify further the magnetic phase diagram of SrCo_2−*x*_Zn*_x_* Fe_16_O_27_, neutron diffraction at various compositions and temperatures should be conducted. This could help clarify whether a conical magnetic structure appears and if this can be stabilized at room temperature. In future work, we hope to reveal the nature of this transition using temperature-dependent neutron diffraction and further investigate if a conical ordering is present and possibly responsible for magnetoelectric coupling (Song *et al.*, 2014 ▸).

## 4. Conclusion

The W-type hexaferrite structures SrCo_2_Fe_16_O_27_, SrCoZnFe_16_O_27_ and SrZn_2_Fe_16_O_27_ were synthesized, and their magnetic symmetries were characterized using a combination of neutron and synchrotron powder diffraction. The two Co-containing compounds have a planar magnetic ordering, described in the magnetic space group *Cm*′*cm*′, while the Zn sample without Co has uniaxial ordering described in *P*6_3_/*mm*′*c*′.

The inclusion of non-collinear terms in the magnetic structural refinement allowed significant improvement in describing the observed data, suggesting that these are present in the magnetic structure of all three samples. Furthermore, the thermomagnetic measurements indicate a magnetic transition below the Curie temperature in the two Co-containing compounds. The presence of Zn lowered both the transition temperature and the Curie temperature. All in all, this shows that both the magnetic ordering and transition temperature can be tuned by the Co/Zn ratio.

## 6. Related literature

For further literature related to the supporting information, see Belov *et al.* (1957 ▸), Campbell *et al.* (2022 ▸) and Opechowski & Guccione (1965 ▸).

## Supplementary Material

Additional background, tables and figures. DOI: 10.1107/S1600576723002133/in5078sup1.pdf

Accompanying CIF and mCIF files. DOI: 10.1107/S1600576723002133/in5078sup2.zip

## Figures and Tables

**Table 1:** Calcination temperatures and times for the five compositions studied

	SrCo_2_Fe_16_O_27_	SrCo_1.5_Zn_0.5_Fe_16_O_27_	SrCoZnFe_16_O_27_	SrCo_0.5_Zn_1.5_Fe_16_O_27_	SrZn_2_Fe_16_O_27_
Temperature (K) and time (h)	1473 K, 2 h	1523 K, 4 h	1523 K, 2 h	1523 K, 1 h	1473 K, 2 h
High-resolution neutron and X-ray measurements	Yes	–	Yes	–	Yes
Thermomagnetic measurements	Yes	Yes	Yes	Yes	Yes

**Table 2:** Overview of refined agreement factors and crystallographic parameters GoF is goodness of fit. The refinement is conducted on four different data sets with the total number of data points being 179 772, divided into 9562 data points (neutron TOF forward scatter bank), 10 036 data points (neutron TOF 90° bank), 10 174 data points (neutron TOF backscatter bank) and 150 000 data points (I11, Diamond multi-analyser crystal setup). The number of independent observations was calculated as described by David (1999 ▸) for SrCo_2_Fe_16_O_27_. This gives a total of 1288 independent observations for the X-ray synchrotron data and 970 independent observations for the neutron data. For the neutron data 250 independent observations were observed at *Q* < 7 Å^−1^ (where 〈*j*_0_〉 for Fe^3+^ is >10%).

	SrCo_2_Fe_16_O_27_	SrCoZnFe_16_O_27_	SrZn_2_Fe_16_O_27_
*R*_wp_ (%), GoF (overall)	6.59, 1.72	6.47, 1.61	6.46, 1.51
*R*_wp_ (%), GoF (backscatter)	6.61, 4.16	4.82, 2.82	5.13, 2.82
*R*_wp_ (%), GoF (90°)	3.80, 2.84	3.39, 2.37	3.78, 2.48
*R*_wp_ (%), GoF (30°)	4.25, 1.22	5.19, 1.14	5.37, 1.13
*R*_wp_ (%), GoF (I11)	11.45, 1.31	11.59, 1.45	11.21, 1.31
Total parameters	208	274	252
Structural parameters WHF	64†	73	39
Magnetic parameters WHF	20†	20	9
Wt% spinel	6.54	7.54 (1.94)‡	4.12
Wt% M-type hexaferrite	–	0.19 (2.10)‡	1.77
Wt% X-type hexaferrite	–	0.52 (2.94)‡	2.67
*a* (Å)	5.89575 (2)	5.89874 (3)	5.908047 (10)
*b* (Å)	10.21042 (4)	10.21562 (5)	5.908047 (10)§
*c* (Å)	32.76195 (7)	32.79282 (9)	32.83467 (6)

† Ratio of independent peaks to parameters for SrCo_2_Fe_16_O_27_ – 28:1 crystal structure and 11:1 magnetic structure.

‡ Phase fractions from X-ray data (I11) given in parentheses.

§ Transformation to rhombohedral axis 5.908047 Å × 3^1/2^ = 10.233038 Å.

**Table 3:** Curie temperatures and suggested transition temperatures, based on the maximum of the derivative

	SrCo_2_Fe_16_O_27_	SrCo_1.5_Zn_0.5_Fe_16_O_27_	SrCoZnFe_16_O_27_	SrCo_0.5_Zn_1.5_Fe_16_O_27_	SrZn_2_Fe_16_O_27_
T_C_ (K)	790	720	700	650	590
T_T_ (K)	555	450	405	265	–
