# Supplementary material for: Controlling the magnetic structure in W-type hexaferrites
Source: J Appl Crystallogr. 2023 Apr 13;56(Pt 3):597–602. doi: 10.1107/S1600576723002133 (PMC10241064; doi:10.1107/S1600576723002133)
Supplement: Supplementary file 1 [file j-56-00597-sup1.pdf]

JOURNAL OF
APPLIED
CRYSTALLOGRAPHY

Volume 56 (2023)

Supporting information for article:

Controlling the magnetic structure in W-type hexaferrites

Mathias I. Mørch and Mogens Christensen

S1. Refinement details

Absorption was modelled as exp(-μ*D_spacing). Weighting between data sets was not modified and kept as $1/\sigma(Y_{obs})^2$. Atomic displacement parameters (ADPs) between Wyckoff sites that split going from $P6_3/mmc \rightarrow Cmcm$ were kept equal. A small difference in lattice parameters was refined between neutron and X-ray data to account for possible temperature differences.

For further details, see the supplied CIF files.

S1.1. Impurities

M-type and X-type hexaferrites and spinel are common and easily overlooked impurities that are hard to avoid. Correctly modelling these allowed for a higher degree of certainty in the remainder of refined parameters. Rietveld refinements showed that all samples had impurities. The high-quality data allowed a description of these. For $SrCoZnFe_{16}O_{27}$ the weight fractions obtained in the data from I11 and SHRPD were not reconcilable. The spinel composition was assumed to be based on the composition of the given W-type hexaferrite. For example, for $SrCoZnFe_{16}O_{27}$, the spinel was assumed to be $Co_{0.5}Zn_{0.5}Fe_2O_4$.

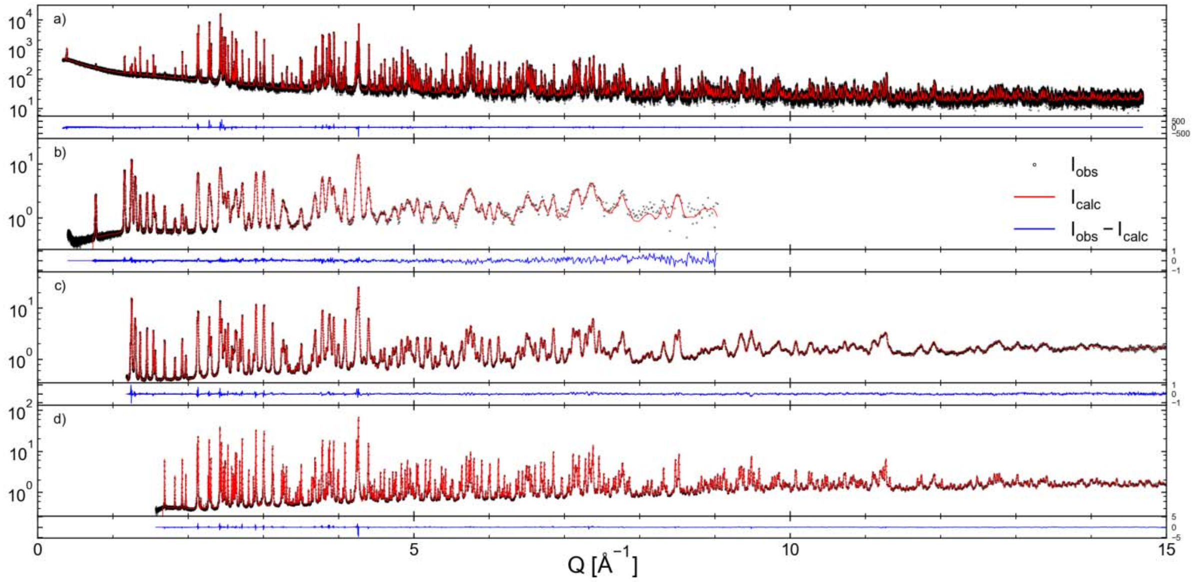

Figure S1 The four powder diffraction patterns of $SrCo_2Fe_{16}O_{27}$ with observed data are given as open circles, calculated as a red line, and shown on a log scale. The difference is shown as a blue line on a linear scale. a) Diamond I11 multi-analyser crystals (MAC), b) SuperHRPD forward scattering bank at 32° c) SuperHRPD perpendicular bank at 90° and d) SuperHRPD backscattering bank at 172°.

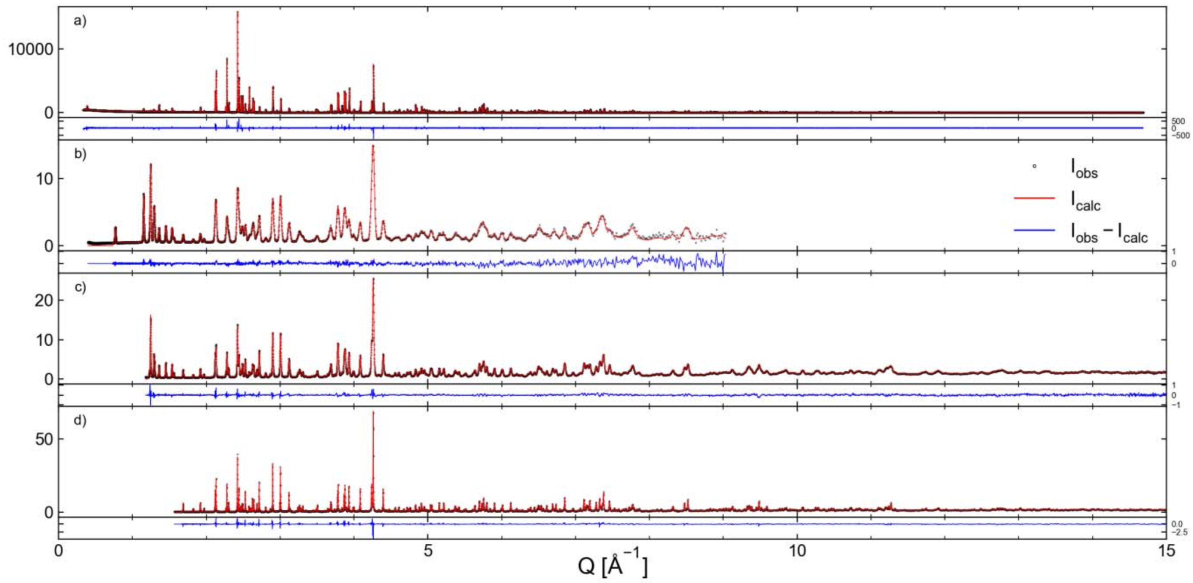

Figure S2 The four powder diffraction patterns of $SrCo_2Fe_{16}O_{27}$ with observed data given as open circles, calculated as a red line, and shown on a linear scale. The difference is shown as a blue line on a linear scale. a) Diamond I11 MAC, b) SuperHRPD forward scattering bank at 32° c) SuperHRPD perpendicular bank at 90° and d) SuperHRPD backscattering bank at 172°.

S1.2. Data-to-parameter ratio

To support the data-to-parameter ratio an example is given for $SrCo_2Fe_{16}O_{27}$ where 208 parameters were refined in total. The data sets of $SrCo_2Fe_{16}O_{27}$ have in total 9562 data points (Neutron TOF forward scatter bank) + 10036 data points (Neutron TOF 90° bank) + 10174 data points (Neutron TOF backscatter bank) + 150000 data points (Synchrotron Multi-analysing crystals) = 179772 data points. Of the 208 parameters, 48 describe the backgrounds, 66 the instrument profiles, and 94 parameters affecting the structure factor. Of the structure factor parameters, 64 are for the $SrCo_2Fe_{16}O_{27}$ structure, while 20 describe the magnetic structure, seven are structural parameters for the $CoFe_2O_4$ impurity and three are for the magnetic structure of $CoFe_2O_4$. The number of independent reflections was calculated following David (1999). For the X-ray data a total of 1288 independent reflections were calculated and for the neutron data 970 independent reflections. Limiting the Q range to $Q < 7$ Å^{-1} (corresponding to $\langle j_0 \rangle$ for Fe^{3+} being > 10%) where the magnetic signal contributes a total of 250 independent reflections were calculated. The ratio between independent reflections and structural parameters is thus for the X-ray data: 1288/71 ~ 18:1. For the neutron data 970/94 ~10:1. For neutron data $Q < 7$ Å^{-1} with only magnetic parameters: 250/23 ~ 11:1. For X-ray and neutron data (Q > 7 Å^{-1}) (1288+970-250)/71 ~ 28:1.

Table S1 $SrCo_2Fe_{16}O_{27}$ Crystal and magnetic structure details

Label	Atom	Wyc	Sym	*x*	*y*	*z*	Occ. (%)	*B* (Å^2)	*Mx* (μ_B)	*My* (μ_B)	*Mz* (μ_B)
Sr1	Sr^{2+}	4*c*	*m*'2*m*'	0	-0.0032(4)	0.25	1	0.616(14)			
Fe1	Fe^{3+}	$8f_{tet}$	*m*'..	0	-0.0015(3)	0.056193(16)	0.9264(19)	0.173(9)		3.997(15)	-1.90(4)
Co1	Co^{2+}	$8f_{tet}$	*m*'..	0	-0.0015(3)	0.056193(16)	0.0736(19)	0.173(9)		2.398(9)	-1.14(3)
Fe2	Fe^{3+}	$8f_{tet}$	*m*'..	0	0.3302(3)	0.593034(15)	0.993(2)	0.235(9)		3.901(14)	-0.07(5)
Co2	Co^{2+}	$8f_{tet}$	*m*'..	0	0.3302(3)	0.593034(15)	0.007(2)	0.235(9)		2.341(8)	-0.04(3)
Fe3_1	Fe^{3+}	$8d_{oct}$	-1	0.25	0.25	0	0.342(6)	0.347(8)	0.11(11)	-4.11(7)	-0.38(6)
Fe3_2	Fe^{3+}	$4b_{oct}$	2'/*m*'..	0	0.5	0	0.878(11)	0.347(8)		-3.96(13)	-0.10(9)
Co3_1	Co^{2+}	$8d_{oct}$	-1	0.25	0.25	0	0.658(6)	0.347(8)	0.06(6)	-2.47(4)	-0.23(4)
Co3_2	Co^{2+}	$4b_{oct}$	2'/*m*'..	0	0.5	0	0.122(11)	0.347(8)		-2.37(8)	-0.06(6)
Fe4	Fe^{3+}	$8f_{oct}$	*m*'..	0	0.3329(3)	0.425905(15)	0.973(2)	0.379(8)		-3.791(13)	-1.20(5)
Co4	Co^{2+}	$8f_{oct}$	*m*'..	0	0.3329(3)	0.425905(15)	0.027(2)	0.379(8)		-2.274(8)	-0.72(3)
Fe5_1	Fe^{3+}	$16h_{oct}$	1	0.7475(2)	0.41991(14)	0.15108(4)	1.000(5)	0.212(5)		-3.04(4)	-0.40(3)
Fe5_2	Fe^{3+}	$8f_{oct}$	*m*'..	0	0.1665(2)	0.15113(7)	0.973(10)	0.212(5)		-3.56(7)	-0.54(4)
Co5_1	Co^{2+}	$16h_{oct}$	1	0.7475(2)	0.41991(14)	0.15108(4)	0.000(5)	0.212(5)		-1.83(2)	-0.239(18)
Co5_2	Co^{2+}	$8f_{oct}$	*m*'..	0	0.1665(2)	0.15113(7)	0.212(5)	0.212(5)		-2.13(4)	-0.32(2)
Fe6	Fe^{3+}	$8f_{oct}$	*m*'..	0	0.3298(3)	0.708354(14)	1.000(2)	0.239(10)		4.108(13)	0.07(4)
Co6	Co^{2+}	$8f_{oct}$	*m*'..	0	0.3298(3)	0.708354(14)	0.000(2)	0.239(10)		2.465(8)	0.04(3)
Fe7	Fe^{3+}	$8f_{tbp}$	*m*'..	0	0.3375(3)	0.24561(4)	0.5000(16)	0.000(18)		-3.236(17)	-4.0(5)
O1_1	O^{2-}	16*h*	1	0.7658(5)	0.4141(3)	0.03672(8)	1	0.540(10)			
O1_2	O^{2-}	8*f*	*m*'..	0	0.1737(5)	0.03630(15)	1	0.540(10)			
O2	O^{2-}	8*f*	*m*'..	0	0.3381(5)	0.53497(3)	1	0.26(2)			
O3_1	O^{2-}	16*h*	1	0.2356(5)	0.2569(4)	0.11128(6)	1	0.264(10)			
O3_2	O^{2-}	8*f*	*m*'..	0	0.4896(5)	0.11002(11)	1	0.264(10)			
O4	O^{2-}	8*f*	*m*'..	0	0.0051(5)	0.11343(3)	1	0.29(2)			
O5_1	O^{2-}	16*h*	1	0.7504(4)	0.0816(3)	0.17923(4)	1	0.214(11)			
O5_2	O^{2-}	8*f*	*m*'..	0	0.8418(3)	0.18276(8)	1	0.214(11)			
O6	O^{2-}	8*f*	*m*'..	0	0.3342(6)	0.31902(3)	1	0.490(19)			
O7_1	O^{2-}	8*g*	..*m*'	0.2711(6)	0.2371(4)	0.25	1	0.617(18)			
O7_2	O^{2-}	4*c*	*m*'2*m*'	0	0.5135(6)	0.25	1	0.617(18)			

Table S2 $SrCoZnFe_{16}O_{27}$ Crystal and magnetic structure details

Label	Atom	Wyc	Sym	x	y	z	Occ (%)	*B* (Å^2)	*Mx* (μ_B)	*My* (μ_B)	*Mz* (μ_B)
Sr1	Sr^{2+}	4*c*	*m*'2*m*'	0	0.0013(7)	0.25	1	0.715(14)			
Fe1	Fe^{3+}	$8f_{tet}$	*m*'..	0	0.0003(4)	0.056351(16)	0.695(4)	0.171(9)		4.32(5)	-2.36(7)
Co1	Co^{2+}	$8f_{tet}$	*m*'..	0	0.0003(4)	0.056351(16)	0.001(6)	0.171(9)		2.59(3)	-1.42(4)
Zn1	Zn^{2+}	$8\mathrm{f}_{tet}$	*m*'..	0	0.0003(4)	0.056351(16)	0.304(7)	0.171(9)			
Fe2	Fe^{3+}	$8f_{tet}$	*m*'..	0	0.3326(4)	0.593312(14)	0.900(4)	0.187(9)		3.96(4)	0.08(5)
Co2	Co^{2+}	$8f_{tet}$	*m*'..	0	0.3326(4)	0.593312(14)	0.000(6)	0.187(9)		2.38(2)	0.05(3)
Zn2	Zn^{2+}	$8f_{tet}$	*m*'..	0	0.3326(4)	0.593312(14)	0.100(8)	0.187(9)			
Fe3_1	Fe^{3+}	$8d_{oct}$	-1	0.25	0.25	0	0.81(3)	0.445(8)	-0.8(2)	-2.88(17)	-0.19(5)
Fe3_2	Fe^{3+}	$4b_{oct}$	2'/*m*'..	0	0.5	0	0.55(5)	0.445(8)		-4.5(4)	0.25(8)
Co3_1	Co^{2+}	$8d_{oct}$	-1	0.25	0.25	0	0.19(3)	0.445(8)	-0.45(13)	-1.73(10)	-0.11(3)
Co3_2	Co^{2+}	$4b_{oct}$	2'/*m*'..	0	0.5	0	0.45(7)	0.445(8)		-2.7(2)	0.15(5)
Zn3_1	Zn^{2+}	$8d_{oct}$	-1	0.25	0.25	0	0.00(4)	0.445(8)			
Zn3_2	Zn^{2+}	$4b_{oct}$	2'/*m*'..	0	0.5	0	0.00(8)	0.445(8)			
Fe4	Fe^{3+}	$8f_{oct}$	*m*'..	0	0.3342(4)	0.425487(14)	1.000(3)	0.311(8)		-3.547(16)	-1.35(4)
Co4	Co^{2+}	$8f_{oct}$	*m*'..	0	0.3342(4)	0.425487(14)	0	0.311(8)		-2.128(9)	-0.81(2)
Zn4	Zn^{2+}	$8f_{oct}$	*m*'..	0	0.3342(4)	0.425487(14)	0.000(3)	0.311(8)			
Fe5_1	Fe^{3+}	$16h_{oct}$	1	0.7475(3)	0.4182(2)	0.15090(3)	1.000(9)	0.216(5)	-0.02(4)	-2.87(3)	-0.41(3)
Fe5_2	Fe^{3+}	$8f_{oct}$	*m*'..	0	0.1641(3)	0.15177(6)	1.000(17)	0.216(5)		-3.44(6)	-0.32(5)
Co5_1	Co^{2+}	$16h_{oct}$	1	0.7475(3)	0.4182(2)	0.15090(3)	0	0.216(5)	-0.01(3)	-1.723(19)	-0.25(2)
Co5_2	Co^{2+}	$8f_{oct}$	*m*'..	0	0.1641(3)	0.15177(6)	0	0.216(5)		-2.06(3)	-0.19(3)
Zn5_1	Zn^{2+}	$16h_{oct}$	1	0.7475(3)	0.4182(2)	0.15090(3)	0.000(9)	0.216(5)			
Zn5_2	Zn^{2+}	$8f_{oct}$	*m*'..	0	0.1641(3)	0.15177(6)	0.000(17)	0.216(5)			
Fe6	Fe^{3+}	$8f_{oct}$	*m*'..	0	0.3312(3)	0.708535(13)	1.000(3)	0.311(9)		4.081(16)	0.13(5)
Co6	Co^{2+}	$8f_{oct}$	*m*'..	0	0.3312(3)	0.708535(13)	0	0.311(9))		2.448(10)	0.08(3)
Zn6	Zn^{2+}	$8f_{oct}$	*m*'..	0	0.3312(3)	0.708535(13)	0.000(3)	0.311(9)			
Fe7	Fe^{3+}	$8f_{tbp}$	*m*'..	0	0.3331(5)	0.24517(3)	0.500(2)	0.000(12)		-3.16(2)	-4.0(4)
O1_1	O^{2-}	16*h*	1	0.7662(6)	0.4145(4)	0.03618(6)	1	0.538(10)			
O1_2	O^{2-}	8*f*	*m*'..	0	0.1771(7)	0.0360312)	1	0.538(10)			
O2	O^{2-}	8*f*	*m*'..	0	0.3302(7)	0.53498(3)	1	0.31(2)			
O3_1	O^{2-}	16*h*	1	0.2313(5)	0.2525(4)	0.11003(5)	1	0.307(11)			
O3_2	O^{2-}	8*f*	*m*'..	0	0.4890(5)	0.11220(10)	1	0.307(11)			
O4	O^{2-}	8*f*	*m*'..	0	-0.0039(7)	0.11364(3)	1	0.29(2)			
O5_1	O^{2-}	16*h*	1	0.7590(5)	0.0801(4)	0.18202(3)	1	0.215(11)			
O5_2	O^{2-}	8*f*	*m*'..	0	0.8322(3)	0.17736(6)	1	0.215(11)			
O6	O^{2-}	8*f*	*m*'..	0	0.3309(7)	0.31926(3)	1	0.26(2)			
O7_1	O^{2-}	8*g*	..*m*'	0.2685(8)	0.2396(5)	0.25	1	0.533(19)			
O7_2	O^{2-}	4*c*	*m*'2*m*'	0	0.51595(8)	0.25	1	0.533(19)			

Table S3 $SrZn_2Fe_{16}O_{27}$ Crystal and magnetic structure details

Label	Atom	Wyc	Sym	*x*	*y*	*z*	Occ (%)	*B* (Å^2)	*Mx* (μ_B)	*My* (μ_B)	*Mz* (μ_B)
Sr1	Sr^{2+}	2*b*	-6*m*'2'	0	0	0.25	1	0.787(12)			
Fe1	Fe^{3+}	$4e_{tet}$	3*m*'.	0	0	0.056509(16)	0.421(2)	0.209(8)			-3.99(4)
Zn1	Zn^{2+}	$4e_{tet}$	3*m*'.	0	0	0.056509(16)	0.579(2)	0.209(8)			
Fe2	Fe^{3+}	$4f_{tet}$	3*m*'.	0.3333333	0.6666667	0.593990(13)	0.860(3)	0.232(9)			-4.511(17)
Zn2	Zn^{2+}	$4f_{tet}$	3*m*'.	0.3333333	0.6666667	0.593990(13)	0.140(3)	0.232(9)			
Fe3	Fe^{3+}	$6g_{oct}$	.2'/*m*'.	0.5	0	0	0.9023(19)	0.337(6)	-0.159(9)	-0.319(18)	2.991(17)
Zn3	Zn^{2+}	$6g_{oct}$	.2'/*m*'.	0.5	0	0	0.0977(19)	0.337(6)			
Fe4	Fe^{3+}	$4f_{oct}$	3*m*'.	0.3333333	0.6666667	0.424857(13)	1.000(3)	0.259(6)			-3.554(13)
Zn4	Zn^{2+}	$4f_{oct}$	3*m*'.	0.3333333	0.6666667	0.424857(13)	0.000(3)	0.259(6)			
Fe5	Fe^{3+}	$12k_{oct}$	.*m*'.	0.16376(3)	0.32752(5)	0.151305(6)	1.000(2)	0.214(4)	0.162(7)	0.324(13)	3.091(5)
Zn5	Zn^{2+}	$12k_{oct}$	.*m*'.	0.16376(3)	0.32752(5)	0.151305(6)	0.000(2)	0.214(4)			
Fe6	Fe^{3+}	$4f_{oct}$	3*m*'.	0.3333333	0.6666667	0.708565(12)	0.966(3)	0.213(8)			-4.353(15)
Zn6	Zn^{2+}	$4f_{oct}$	3*m*'.	0.3333333	0.6666667	0.708565(12)	0.034(3)	0.213(8)			
Fe7	Fe^{3+}	$4f_{tbp}$	3*m*'.	0.3333333	0.6666667	0.24507(3)	0.5	0.004(12)			3.786(14)
O1	O^{2-}	12*k*	.*m*'.	0.17811(5)	0.35622(11)	0.035224(12)	1	0.417(8)			
O2	O^{2-}	4*f*	3*m*'.	0.3333333	0.6666667	0.53490(2)	1	0.376(15)			
O3	O^{2-}	12*k*	.*m*'.	0.48905(6)	-0.02189(12)	0.110539(13)	1	0.383(8)			
O4	O^{2-}	4*e*	3*m*'.	0	0	0.11473(2)	1	0.380(15)			
O5	O^{2-}	12*k*	.*m*'.	0.83720(6)	0.67441(12)	0.180521(11)	1	0.367(8)			
O6	O^{2-}	4*f*	3*m*'.	0.3333333	0.6666667	0.31943(2)	1	0.462(17)			
O7	O^{2-}	6*h*	*mm*'2'	0.51523(8)	0.03045(17)	0.25	1	0.575(12)			

S2. Symmetry

$SrCo_2Fe_{16}O_{27}$ and $SrCoZnFe_{16}O_{27}$ were described in the orthorhombic space group *Cm'cm'*. Figure S3 shows the relation between this and the hexagonal space group $P6_3/mmc$ for $SrZn_2Fe_{16}O_{27}$.

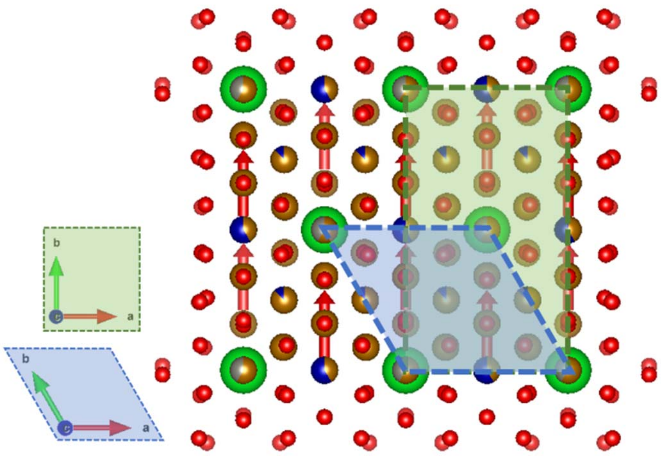

Figure S3 The relation between the hexagonal $P6_3/mmc$ (blue) and orthorhombic *Cmcm* (green) space groups for $SrZn_2Fe_{16}O_{27}$ is illustrated.

S2.1. Magnetic space groups

For the magnetic space group $P6_3/mm'c'$ the serial numbers are respectively: Belov-Neronova-Smirnova (BNS) (Belov *et al.*, 1957) 194.270, where 194: the space group number of $P6_3/mmc$ and 270: the 270th magnetic space group of the crystal system. Opechowski-Guccione (OG) (Opechowski & Guccione, 1965) 194.8.1501, where 194 is the space group as above, 8: The 8th magnetic space group of $P6_3/mmc$, 1501: The 1501st magnetic space group. The new UNI symbols (Campbell et al., 2022) for magnetic space groups do not use these serial numbers. The magnetic space group symbol $P6_3/mm'c'$ is common to all three naming conventions.

If mGM6+ was the only magnetic distortion mode active in $SrCo_2Fe_{16}O_{27}$ and $SrCoZnFe_{16}O_{27}$, *Cmc'm'* describes this. The common subgroup with $P6_3/mm'c'$ is then $C2'/c'$ and the group-subgroup relation for these, similar to the one shown in Fig. 2, is shown here in Figure S4.

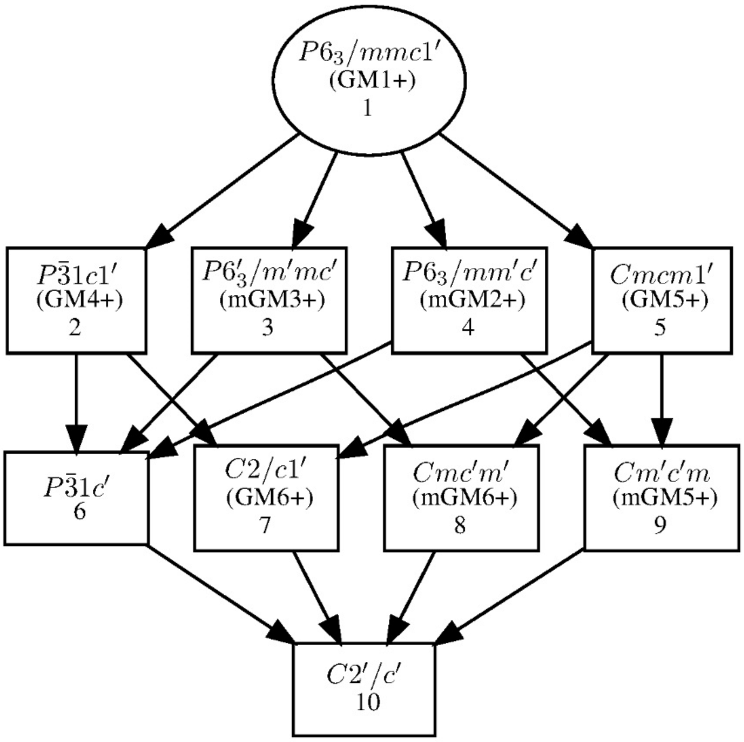

Figure S4 Subgroup relation of *Cmc'm'* and *P*6₃/*mm'c'*

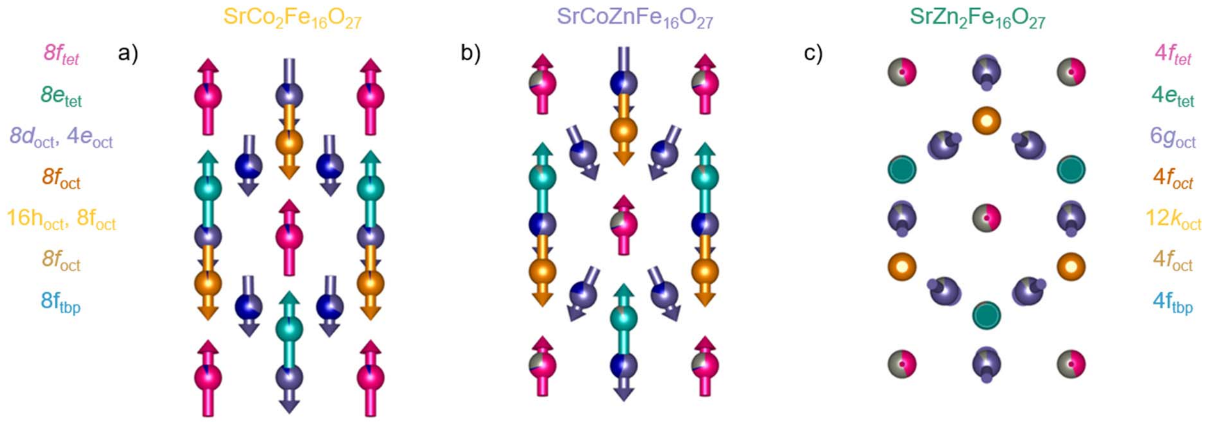

Figure S5 Comparison of the canting on the $8d_{oct}$ and $6g_{oct}$ site between b) $SrCoZnFe_{16}O_{27}$ and c) $SrZn_2Fe_{16}O_{27}$, while absent in a) $SrCo_2Fe_{16}O_{27}$. The unit cell shown from c = 0:0.125, Oxygen atoms omitted for clarity.

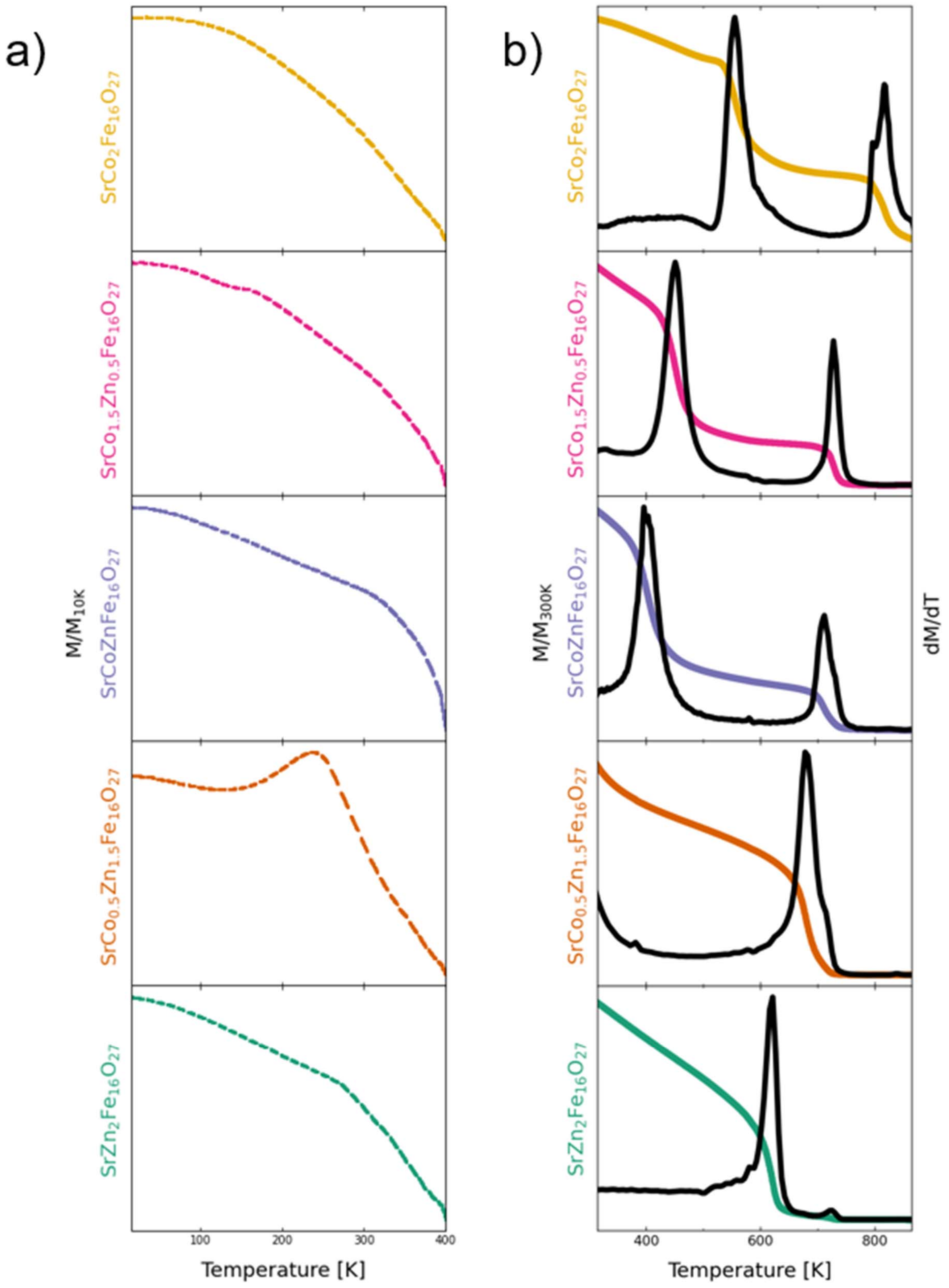

Figure S6 Thermomagnetic measurements of $SrCo_{2-x}Zn_xFe_{16}O_{27}$ a) from 10 to 400 K and b) from 325 to 875 K. The magnetization (in colour) and the derivative thereof (in black) are normalized for easy comparison.
